# The Effect of Power Asymmetries on Cooperation and Punishment in a Prisoner’s Dilemma Game

**DOI:** 10.1371/journal.pone.0117183

**Published:** 2015-01-28

**Authors:** Jonathan E. Bone, Brian Wallace, Redouan Bshary, Nichola J. Raihani

**Affiliations:** 1 CoMPLEX, University College London, London, United Kingdom; 2 Department of Economics, University College London, London, United Kingdom; 3 Institut de Biologie, Eco-Ethologie, Université de Neuchâtel, Neuchâtel, Switzerland; 4 Department of Genetics, Evolution and Environment, University College London, London, United Kingdom; Universidad Carlos III de Madrid, SPAIN

## Abstract

Recent work has suggested that punishment is detrimental because punishment provokes retaliation, not cooperation, resulting in lower overall payoffs. These findings may stem from the unrealistic assumption that all players are equal: in reality individuals are expected to vary in the power with which they can punish defectors. Here, we allowed strong players to interact with weak players in an iterated prisoner's dilemma game with punishment. Defecting players were most likely to switch to cooperation if the partner cooperated: adding punishment yielded no additional benefit and, under some circumstances, increased the chance that the partner would both defect and retaliate against the punisher. Our findings show that, in a two-player game, cooperation begets cooperation and that punishment does not seem to yield any additional benefits. Further work should explore whether strong punishers might prevail in multi-player games.

## Introduction

Punishment involves an individual paying a cost in order to inflict harm on a cheat [1,2]. This investment can be recouped if the punished individual—or a bystander—behaves more cooperatively in subsequent encounters [1,3,4]. Although people are apparently willing to pay to harm cheats [5–15] and even derive subjective pleasure from doing so [16,17], consensus is still lacking over whether punishment is effective at promoting cooperation [9,10,15,18–22]. Although punishment increases payoffs in long-run encounters [23], others have found no benefit to punishers [5,6,13], or that punishment reduces the payoffs of the punisher or their group [8,9,12,24,25]. Punishment is especially likely to reduce payoffs to punishers where it provokes retaliation, rather than cooperation, because, as well as the cost of inflicting the punishment, the punisher incurs an additional cost of being punished (e.g. [24,26–28]). Indeed, when retaliation is possible, punishment has been shown to reduce payoffs even in long-run encounters [29]. Based on these findings, it has been argued that rewards are more effective at sustaining cooperation [30] and that punishment is unlikely to have evolved as a cooperation-enforcing mechanism [24].

Some of the puzzling findings regarding punishment might stem from the assumption in several studies (e.g. [8,9,24]) that all players are equal. In reality individuals are expected to often vary in power or resource holding potential, such that some players are able to inflict a greater cost on the partner than the partner is able to reciprocate. In fact, it has been suggested that punishment is most likely to operate down a dominance hierarchy, with dominants punishing subordinates who are, in turn, unlikely to retaliate [1,2,31–33]. For example, experiments using cleaner fish (*Labroides dimidiatus*), have shown that males (the larger sex) punish females that cheat during joint inspections of model clients but—apparently due to the size difference—females never retaliate or punish males [3,34]. Similarly, a recent field study revealed that both human males and females were more likely to confront females than males for dropping litter. Moreover, fear of retaliation was the most common reason survey respondents gave for not confronting litterers [35]. Power asymmetries might therefore be expected to fundamentally affect the outcome of human interactions involving punishment but have received relatively little attention in comparison to other asymmetries (e.g. in cost of contribution [36,37], or benefits derived from [38–40] a public good). One study which did explore the effect of power asymmetries showed that in the setting of a public goods game, strong players contributed similar amounts as weak players but also punished more and received higher payoffs than weak players [41]. Although punishment raised the contributions of low contributors, this study did not explore whether the effectiveness of punishment was affected by power asymmetries, as would be expected. Furthermore, players were not informed which of their peers punished them; thereby preventing retaliation. Thus, the effect of power asymmetries on punishment use and effectiveness in humans remains poorly understood.

Here, we incorporated power asymmetries into a two-player iterated prisoner's dilemma (IPD) game [42] with punishment (similar to [24]) in order to explore how asymmetries affected the use and effectiveness of punishment in a setting where retaliation was possible. Asymmetries were incorporated into the game by allowing strong players to interact with weak players. As in, Nikiforakis et al. (2010) [41], investing in punishment cost all players the same amount but strong players could inflict greater damage through punishing than weak players. Several studies have shown that punishment which inflicts greater damage is used more frequently [8,11,12,41,43] and is more effective at promoting cooperation [8,11,22,43] than milder punishment. We predicted that weaker players would be more cooperative in asymmetric than symmetric games, particularly after being punished for defecting. We expected weak players would rarely punish in asymmetric games and that power asymmetries would reduce the likelihood that weaker players retaliate in response to punishment, thereby decreasing the cost associated with punishment for stronger players [1,2]; but see [40].

It was envisaged that by these mechanisms, punishment use for strong players could promote cooperation more effectively in asymmetric games than in symmetric games. We chose to focus on the behavioural consequences of punishment, rather than the payoffs accruing to punishers. In artificial laboratory settings, individual payoffs are necessarily determined by the (largely) arbitrary costs and benefits associated with the different actions available to players in the game. In addition, the time horizon of the interaction, which is again largely determined arbitrarily by the experimenter, can have a fundamental bearing on whether punishment is found to improve payoffs: punishment is least likely to be beneficial in short-run interactions [23]. Since the net benefit of punishment in real-world settings must emerge from its ability to deter partners (or bystanders [4]) from defecting, we suggest that quantifying the effect of punishment on targets' behaviour, rather than the total payoffs accruing to the punisher, is more important for understanding the functional basis of punishment.

## Methods

### 2.1 Experimental protocol

This research was approved by the University College London ethics board (project number 3720/001). All subjects remained anonymous so informed consent about the use of personal data was deemed unnecessary and was therefore waived by the University College London ethics board. The experiment took place over six sessions, one in May 2012, one in Nov 2012, two in March 2013 and two in April 2014 in the experimental laboratory in the Department of Economics, University College London. The lab consists of twenty computers, which are visually partitioned. A total of 120 participants (63 women, 57 men, mean age ± se = 22.0 ± 0.39 years) were recruited to play an IPD game with a punishment option Players interacted anonymously in pair-wise encounters by means of computer screens using the z-Tree [44] software. All players were paid a £5.00 show-up fee. Each player played two games and their final score was summed over both games and multiplied by £0.06 to determine additional earned income. Thus, one game unit corresponded to £0.06. To allow for negative incomes while maintaining the £5.00 show-up fee, all players began each game with 75 units (£4.50) to play with. The average payment per player was £16.69 and the average session length was 90 minutes. Prior to the experiment, each player was given written instructions about the game structure and required to answer nine comprehension questions to verify their understanding of the game (see electronic supplementary material for experimental instructions and questions). The average score from the comprehension questions was 95%. Players were informed of the correct answers after the test.

The IPD game lasted 50 rounds. To avoid end effects [45], players were told that each game would last between 20 and 100 rounds. Players’ behaviour did not change abruptly towards the end of the game (Figure A in S2 Supporting Information), indicating that end effects were absent. As in Dreber et al. (2008) [24], we constructed our IPD such that cooperation implied paying a cost for the other person to receive a benefit, whereas defection implied taking something away from the other person (see Table 1).

**Table 1:** Payoffs accruing to (Player 1, Player 2) in step 1.

		Player 2	
		Cooperate	Defect
Player 1	Cooperate	(1, 1)	(-2, 3)
	Defect	(3, -2)	(0, 0)

Players were randomly split into two types: weak and strong. Weak players punished with a 1:1 fee to fine ratio, meaning that if they chose to punish their partner it would cost them one unit and it would also cost their partner one unit. Strong players punished with a 1:4 fee to fine ratio (as in [24]), meaning that punishing their partner would cost them one unit but it would cost their partner four units. Each player played one game with a partner of the same type as themselves (symmetric) and one game with a partner of a different type (asymmetric). The order in which players played symmetric and asymmetric games was counter-balanced.

In this study we were interested in the effects of power asymmetries on punishment, rather than on coercive behaviour. Coercion is similar to punishment since they are both aggressive behaviours that can induce cooperation from the target. However, coercion differs from punishment because unlike punishment which is typically aimed at producing mutual cooperation, coercion forces the target into a position where they would do better if they could avoid interacting with the aggressor altogether, but are constrained to do so [2].

To ensure that weak partners were not being coerced into cooperating with a defecting strong partner (e.g. [46]), players could choose to opt out of the current round (rather than cooperating or defecting). If either player chose to opt out, step two of the current round was skipped and the next round then began as normal. After both players made their choice, they were shown their own and their partner’s choice and each player's payoffs from this step.

Each round of the game was split into two steps. In step one, both players simultaneously chose between the options of “cooperate”, “defect”, or “do not participate in this round” (opt out). In step two, players were given the option of whether or not to punish the partner. Thus, unlike Dreber et al. (2008) [24], choosing to punish in our game did not imply that players must also forego the option to cooperate in that round. At the end of step 2, players were shown their own and their partner's choice and payoff from step 2, as well as the cumulative payoffs for both players for that round and their own total payoff (summed over all rounds). To prevent negative incomes, if a player’s total payoff became zero or below, they were bankrupt and both they and their partner were unable to take part in the remaining rounds of the game. Only three players went bankrupt during the game (two were weak players in asymmetric games and one was a strong player in a symmetric game). At the end of the first game, players were presented with the final scores and then randomly re-matched for the second game.

In order to avoid framing effects, neutral language was used. Player types “weak” and “strong” were replaced with “type 1” and “type 2”; “cooperate” and “defect” were replaced with “option A” and “option B”; and “punish” and “don’t punish” were replaced by “option C” and “option D”. After both games had finished, all subjects were required to fill in a questionnaire to provide demographic information (see ESM for questions and demographic data).

### 2.2 Analyses

We asked how asymmetries affected (i) players' tendency to cooperate and (ii) players' tendency to punish their partner’s defection. Previous work has shown that cooperators are more likely than defectors to use punishment [43]. Thus, in analysis (ii), we controlled for whether or not the player cooperated (see Table 2 for a full list of all models with response and explanatory terms).

**Table 2:** Explanatory terms: player type is a 2-level factor with levels 'weak' and 'strong'; game type is a 2-level factor with levels 'symmetric' and 'asymmetric'; player cooperated is a 2-level factor describing whether the player cooperated or defected in the current round; partner punished is a 2-level factor describing whether or not the player was punished by their partner in the previous round; partner cooperated is a 2-level factor describing whether the partner cooperated or not in the previous round.

Model	Question	Response term	Explanatory terms	n for analysis
(i)	Do asymmetries affect cooperation?	Player defected (0)	Player type (strong)	9610 rounds
		Player cooperated (1)	Game type (asymmetric)	
			Player type x game type	
(ii)	Do asymmetries affect punishment?	Player did not punish defecting partner (0)	Player type (strong)	2173 rounds
		Player did punish defecting partner (1)	Game type (asymmetric)	
			Player cooperated (yes)	
			All 2-way interactions and the 3-way interaction	
(iii)	Do asymmetries affect whether punishment promotes cooperation?	Player continued to defect (0)	Game type (asymmetric)	
(a.1)	Player is weak	Player switched to cooperate (1)	Partner Punished (yes)	936 rounds
(b.1)	Player is strong & partner defected		Partner cooperated (yes)	476 rounds
(b.2)	Player is strong & partner cooperated		All 2-way interactions and the 3-way interaction (Partner cooperated and the 3-way interaction are only included in model 1.1)	236 rounds
(iv)	Do asymmetries affect whether punishment provokes retaliation?	Player did not punish cooperative partner (0)	Player type (strong)	
(a)	Partner is weak	Player did punish cooperative partner (1)	Partner punished (yes)	N/A
(b)	Partner is strong		Player type x Partner punished	176 rounds
(v)	Do asymmetries affect whether punishment provokes opting out?	Player did not opt out (0)	Player type (strong)	2157 rounds
		Player opted out (1)	Game type (asymmetric)	
			Partner punished (yes)	
			Partner cooperated (yes)	
			All 2-way interactions and 3-way interactions	

Next, we asked how players responded to being punished. We asked whether punishment affected the likelihood that a player would subsequently (iii) cooperate (iv) retaliate or (v) opt out in the next round in both symmetric and asymmetric games. We compared the likelihood that players cooperated and retaliated in round *n + 1* after having been punished (or not) in round *n*. We classed a player as retaliating if they punished a cooperative partner in round *n + 1*, having been punished by that partner in round *n*. For analyses (iii)—(v), data were restricted to instances where players had defected in round *n* (i.e. the effect of antisocial punishment on players' behaviour was not measured).

Players' behaviour in round *n + 1* could also be affected by whether their partner cooperated or defected in round *n*. Therefore, in analyses (iii) & (v), we controlled for whether or not the partner cooperated in round *n*. However, since opportunities to retaliate were relatively rare (N = 107 opportunities for retaliation), in analysis (iv), we did not have sufficient data to control for the effects of whether or not the partner cooperated in round *n* (see Table 2 for a full list of all models with response and explanatory terms).

For analysis (iii), initially a three-way interaction was a component of the best model (Table B in S2 Supporting Information) so separate models were produced for weak and strong players for ease of interpretation (Table 2). A three-way interaction was also a component of the best model when data were restricted to strong players so separate models were produced for strong players with defecting partners and strong players with cooperating partners (Table 2). For analysis (iv), a model was only produced for players with strong partners because we did not record any instances of retaliation against weak partners (Table 2; Table 3).

**Table 3:** The proportion of players who cooperated (± SE), the proportion of rounds in which players cooperated / defected / opted out, the proportion of instances in which the partner defected (D) / cooperated (‘antisocial punishment’) that cooperating players & defecting players responded with punishment and the proportion of instances in which players punished their partner in round *n + 1* (when the partner cooperated) following being punished for defecting in round *n* (‘retaliation’).

	Weak players		Strong Players	
	Symmetric	Asymmetric	Symmetric	Asymmetric
Cooperated	0.70 ± 0.01	0.77 ± 0.01	0.84 ± 0.01	0.78 ± 0.01
Punished partner for D (Player cooperated)	0.25 ± 0.04	0.31 ± 0.04	0.39 ± 0.04	0.59 ± 0.04
Punished partner for D (Player defected)	0.07 ± 0.01	0.11 ± 0.02	0.23 ± 0.03	0.27 ± 0.02
Antisocial punishment	0.03 ± 0.00	0.04 ± 0.00	0.05 ± 0.00	0.09 ± 0.01
Opted out	0.12 ± 0.06	0.19 ± 0.01	0.08 ± 0.00	0.04 ± 0.00
Retaliation	0 ± 0	0.21 ± 0.06	0.24 ± 0.07	0 ± 0

All proportions (except for ‘opted out’) exclude rounds in which either player opted out.

### 2.3 Statistical methods

Data were analysed using R version 2.15.2 [47]. Generalised linear mixed models (GLMMs) with binomial error structure were used for analyses (i)–(v). For comparison, all models were fit with both logit and probit link functions. The qualitative results (sign, significance, order of magnitude) were the same for both types of models. In addition, all models were at least as good a fit with the logit link rather than the probit link, if not better (based on AICc). For this reason we report only the results from logit regressions. To determine the importance of explanatory terms in our models, we used an information theoretic approach with model averaging [48]. GLMMs allow repeated measures to be fitted as random terms, thus controlling for their effects on the distribution of the data. Player identity was included as a random term in all models produced. For all analyses, we excluded rounds where either player was bankrupt. For each analysis we initially generated a global model. Following the specification of the global model, explanatory input variables were centred by subtracting their mean [49]. Centring of input variables allows averaging over models that include different interaction terms [48]. Sub-models were derived from the global model using the package MuMIn [50]. The degree of support for each sub-model was calculated using Akaike's Information Criterion corrected for small sample sizes (AICc) [51]. The subset of top models was identified by taking the best model (the model with the lowest AICc value) and any models within 2AICc units of the best model. Each model in the top set were given Akaike weights, representing the probability that the given model is the true model (compared to other candidate models in the set) [52]. We computed the average parameter estimate (‘effect size’) and relative importance of each term from the top model set. The importance of a term can be thought of as the probability that the given term is a component of the best model; it is calculated by summing the Akaike weights of all models that include the term in question [52]. We only present the effect sizes from the top models (see ESM for effect sizes for analyses (iv) and (v)).

## Results

**Fig 1:**
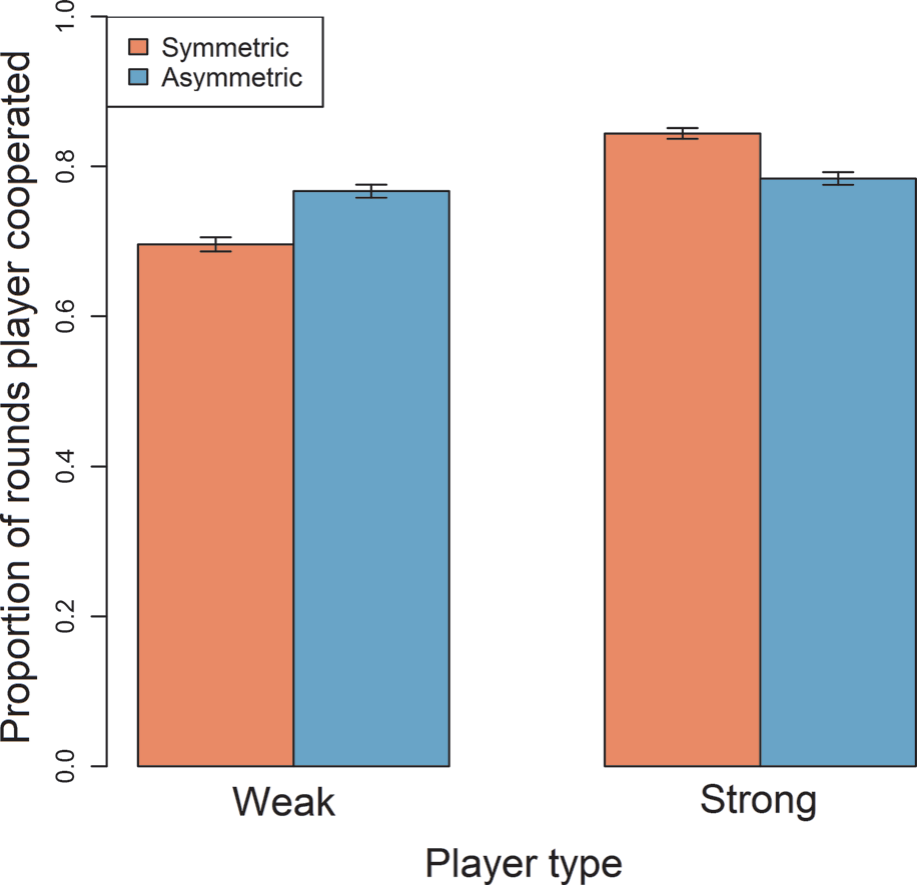
Barplot showing the proportion of rounds which players cooperated in symmetric (red bars) and asymmetric (blue bars) games according to whether they were weak or strong. Data exclude rounds where either player opted out or was bankrupt. Error bars represent standard errors. Plots are generated from raw data.

Cooperation was relatively common in all game types and strong players were typically more cooperative than weak players (Table 3). Cooperation levels varied with game type: although weak players were more cooperative in asymmetric games than in symmetric games, strong players were less cooperative in asymmetric games than in symmetric games (Table 3; Table 4; Fig. 1). Thus, both weak and strong players were most likely to cooperate if their partner was strong.

**Table 4:** Effect sizes, unconditional standard errors, confidence intervals and relative importance for parameters included in the top models for the binary response term encoding whether or not players cooperated in each round of the game (player defected = 0, player cooperated = 1).

Parameter	Effect size	SE	Confidence Interval	Importance
Intercept	1.41	0.21	(0.99, 1.81)	
Player type (strong)	1.18	0.41	(0.36, 2.00)	1.00
Game type (asymmetric)	-0.1	0.06	(-0.21, 0.03)	1.00
Player type x Game type	-0.99	0.12	(-1.23, -0.75)	1.00

**Fig 2:**
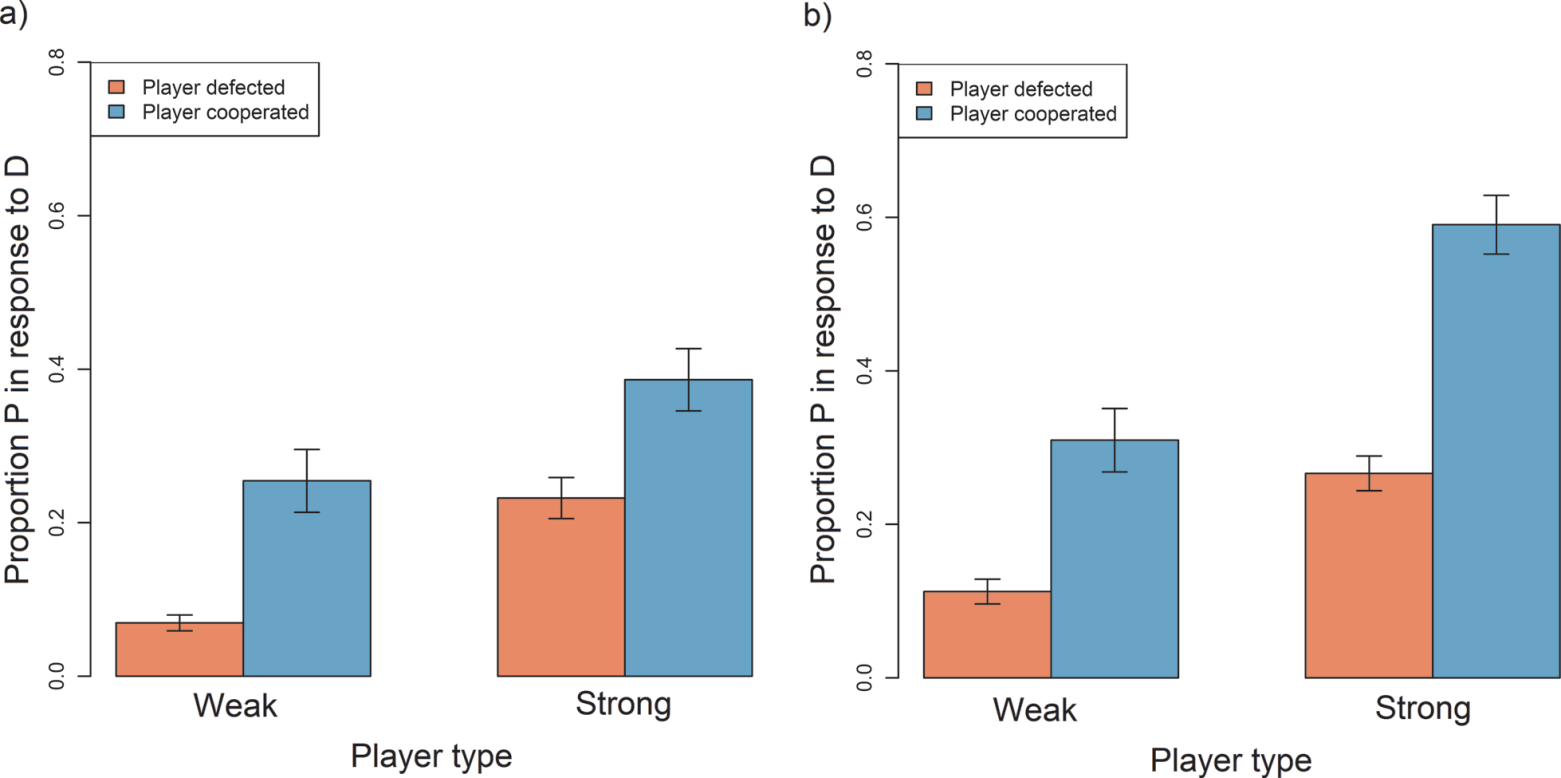
Barplot showing the proportion of their partner's defection that players punished in a) symmetric and b) asymmetric games according to whether they were weak or strong and whether they cooperated or defected them self. Data were restricted to instances in which the partner defected in the previous round, excluding rounds where either player opted out or was bankrupt. Error bars represent standard errors. Plots are generated from raw data.

In general, players were most likely to punish a defecting partner if they themselves had cooperated; the magnitude of this effect was larger for weak players than strong players (Table 3; Table 5; Fig. 2). Strong players were typically more likely to punish than weak players (Table 3; Table 5; Fig. 2). Punishment was used most often—by players of both types—in asymmetric rather than symmetric games (Table 3; Table 5; Fig. 2).

**Table 5:** Effect sizes, unconditional standard errors, confidence intervals and relative importance for parameters included in the top models for the binary response term encoding whether or not players punished their partners for defecting (player did not punish defecting partner = 0, player did punish defecting partner = 1).

Parameter	Effect size	SE	Confidence Interval	Importance
Intercept	-1.81	0.18	(-2.16, -1.47)	
Player type (strong)	1.15	0.35	(0.88, 2.22)	1.00
Game type (asymmetric)	0.88	0.19	(0.51, 1.26)	1.00
Player cooperated (yes)	1.29	0.16	(0.97, 1.61)	1.00
Game type x Player cooperated	0.47	0.29	(-0.11, 1.04)	1.00
Player type x Player cooperated	-0.75	0.31	(-1.37, -0.14)	0.65
Player type x Game type	0.16	0.36	(-0.55, 0.87)	0.19

**Fig 3:**
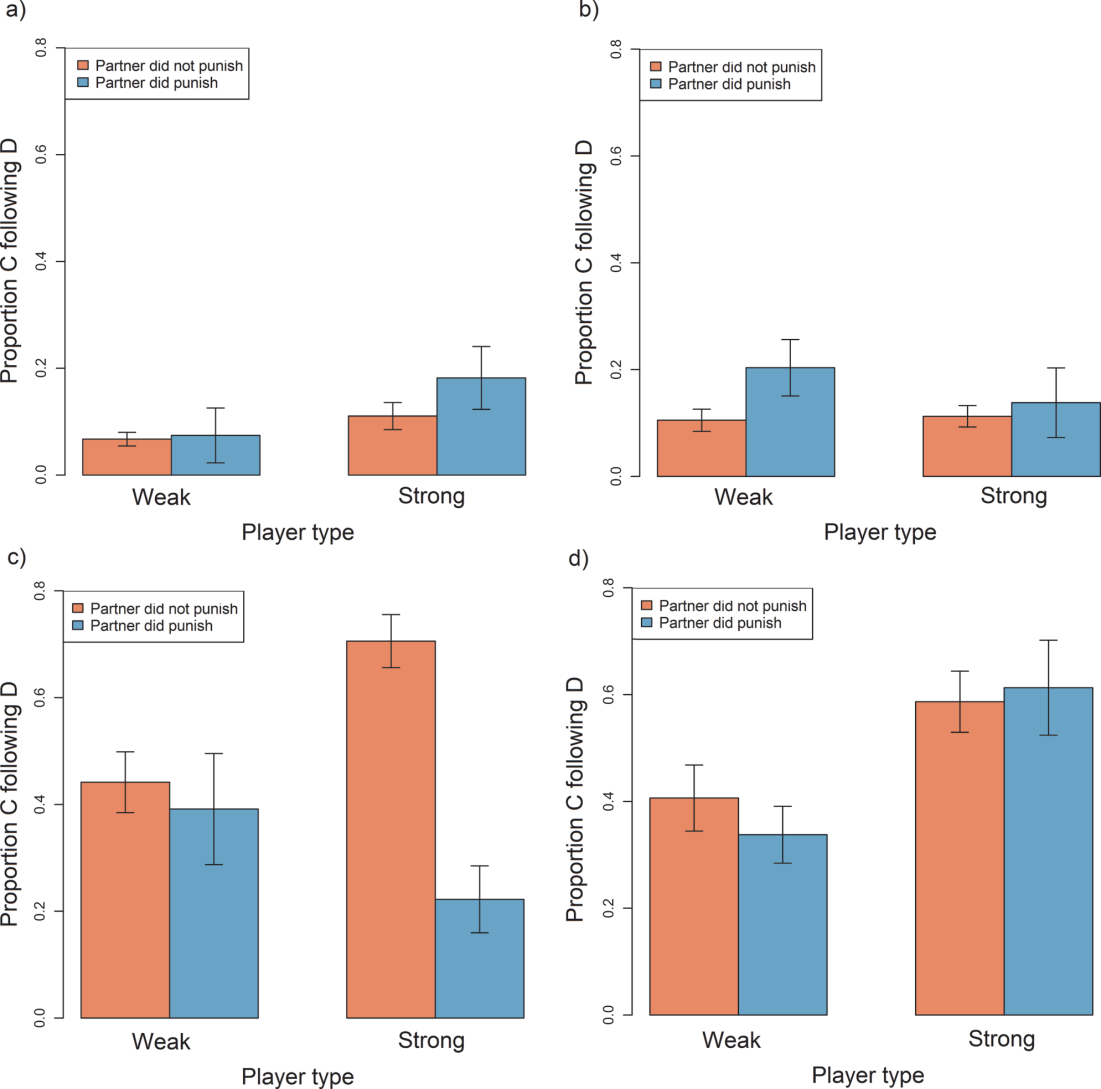
Barplot showing the proportion of instances in which players cooperated following defecting in the previous round, according to whether they were weak or strong and whether they were punished by their partner in the previous round. Data is shown for players in **a)** symmetric games where the partner defected in the previous round **b)** asymmetric games where the partner defected in the previous round **c)** symmetric games where the partner cooperated in the previous round and **d)** asymmetric games where the partner cooperated in the previous round. Data were restricted to instances in which the player defected in the previous round, excluding rounds where either player opted out or was bankrupt. Error bars represent standard errors. Plots are generated from raw data.

If the punisher had also defected (i.e. punishment was 'unjustified' or 'hypocritical'), then punishment had no meaningful effect on the target’s propensity to cooperate in the next round (Table 6; Fig. 3). The effect of being punished by a cooperative partner ('justified punishment') produced more variable outcomes on the target's behaviour. Where justified punishment was aimed at weak players, it produced no discernible effect on subsequent tendency to cooperate (cooperate if not punished = 0.43 ± 0.04; versus if punished = 0.35 ± 0.05). Justified punishment aimed at strong players produced different outcomes. When the punishment was administered by a weak partner, it had no discernible effect on the strong player's cooperative behaviour in the next round (cooperate if not punished = 0.59 ± 0.06; versus if punished = 0.61 ± 0.09). However, in a strong-symmetric game, justified punishment actually reduced the tendency of the player to cooperate in the next round (cooperated if not punished = 0.71 ± 0.05; versus if punished = 0.22 ± 0.06; Table 6; Fig. 3). Rather than responding to punishment, defecting players were more likely to subsequently cooperate when the partner had cooperated (compared to when the partner had also defected) (cooperate if partner cooperated = 0.48 ± 0.02; versus if partner defected = 0.1 ± 0.01). Thus, cooperative behaviour from the partner appeared to be sufficient to convert defectors to cooperators, and punishment in addition to cooperation did not yield any additional benefits.

**Table 6:** Effect sizes, unconditional standard errors, confidence intervals and relative importance for parameters included in the top models investigating players' responses to being punished for defecting in the previous round (player continued to defect = 0, player switched to cooperating = 1).

Player type	Partner cooperated	Parameter	Effect size	SE	Confidence Interval	Importance
Weak	NA	Intercept	1.85	0.19	(-2.21, -1.48)	
		Game type (asymmetric)	0.66	0.28	(0.11, 1.22)	1.00
		Partner punished (yes)	0.38	0.34	(-0.29, 1.05)	1.00
		Partner cooperated (yes)	1.86	0.22	(1.42, 2.30)	1.00
		Game type x Partner cooperated	-1.28	0.47	(-2.20, -0.36)	0.72
		Punished punished x Partner cooperated	-0.88	0.50	(-1.85, 0.10)	0.45
		Game type x Partner punished	0.57	0.58	(-0.57, 1.71)	0.27
						
Strong	No	Intercept	-2.16	0.26	(-2.68, -1.64)	
		Game type (asymmetric)	-0.15	0.42	(-1.00, 0.71)	0.22
		Partner punished (yes)	0.17	0.42	(-0.66, 1.00)	0.22
						
	Yes	Intercept	0.45	0.35	(-0.23, 1.22)	
		Game type (asymmetric)	0.01	0.46	(-0.92, 0.89)	1.00
		Partner punished (yes)	-0.87	0.46	(-1.78, 0.02)	1.00
		Game type x Partner punished	2.43	0.87	(0.77, 4.20)	1.00

Neither weak nor strong players ever retaliated against a weak partner (Table 3). However, weak and strong players did and were equally likely to retaliate in response to punishment from strong partners (Table 3; Table C & Figure B in S2 Supporting Information). In general, weak players opted out more often than strong players (Table 3; Table D in S2 Supporting Information). In addition, both weak and strong players were more likely to opt out if their partner was strong and if they were punished in the previous round (effect size = 0.94, 95% CI = (0.55, 1.32); Table D in S2 Supporting Information). Players were also less likely to opt out if their partner cooperated, rather than defected, in the previous round (effect size = -1.04, 95% CI = (-1.51, -0.57); Table D in S2 Supporting Information).

## Discussion

Previous studies have suggested that punishment use is detrimental because it induces retaliation rather than cooperation [24,26,27]. However, previous studies have typically not accounted for asymmetries between players, which might affect how targets respond to being punished. In this two-player game, punishment did not promote cooperation under any circumstances. Moreover, punishment from strong players provoked (i) further defection and (ii) retaliation from the target. Therefore, punishment carried both the cost of inflicting the punishment itself and the cost associated with retaliation (for strong players) but did not confer the benefits of increased cooperation. We discuss reasons why our findings may not have matched the theoretical predictions below.

As expected, punishment was most often directed from cooperating players at defecting partners and strong players were generally more likely to punish than weak players. While these results supported our initial predictions, we had other findings that were more puzzling. First, weak players were more likely to punish in asymmetric games than in symmetric games, which is the opposite pattern to that which we predicted. Second, players seemed to respond more to the cooperative decision of the partner than to the punitive action when deciding how to behave in the next round. In situations where the partner cooperated, there was no additional positive effect of punishment on the propensity of the target to cooperate and, in fact, in some cases the punishment made the target more likely to defect. Third, while strong punishers frequently encountered retaliation from the target, we did not record a single instance of retaliation against a weak punisher. These results are clearly counter to our predictions that punishment would operate down a dominance hierarchy and be most likely to promote cooperation when directed from strong players at weak targets. In part these findings might stem from the fact that cooperating and defecting were specified as binary, all-or-nothing responses in this study, rather than continuous variables. Thus, a player that switched to cooperation (in response to the partner's cooperation) could not, by definition, increase their investment even more in response to being punished. This is unlike the situation with cleaner fish, where, although defecting is a binary outcome (bite client / do not bite client) investment can be modelled as a continuous variable (duration of time 'cooperating' by removing ectoparasites) [33]. In other real world settings it is debatable whether cooperation should be modelled as an all-or-nothing response or instead as a continuous variable [53–56]. Had we specified cooperation as a continuous variable in this study, we may have been more likely to measure a meaningful effect of punishment on the target's subsequent cooperation. This remains an important avenue for further exploration.

Another possibility is that the effectiveness of punishment varies between two-player games and multi-player games. Previous theoretical and empirical studies [24,30] have argued that in two player games selection favours strategies that cooperate conditionally rather than paying to punish cheats. Our data seem to support these arguments, even when player asymmetries are included in games. Although conditional cooperation is successful in two-player games, such strategies might be less effective in multi-player games because defecting in response to a defector harms the cooperative partners in the group as well as the cheats [2]. Thus, although asymmetries did not allow punishment to promote cooperation in this two-player game, they may be more effective in a multi-player game (e.g. [57]). Furthermore, punishment is likely to be most effective at promoting cooperation where the possibility for retaliation is reduced, for example when punishment is administered by a legitimate authority [58] or when it is administered jointly by several group members [59].

While these explanations might help us to understand why punishment did not promote cooperation, it is less clear why punishment from strong, but not weak, players was actually detrimental—being more likely to provoke both retaliation and defection. It has been suggested that the moral legitimacy of punishment is an important determinant of how the target is expected to respond [18,60]. According to Fehr & Rockenbach (2003) [18], punishment may be perceived as being morally illegitimate if it is associated with selfish or greedy (rather than altruistic) intentions. In this way, punishment that increases the payoffs of the punisher, relative to the target (as punishment from strong players in our study did), may be interpreted as a competitive act and therefore perceived as morally illegitimate. It has been argued that morally illegitimate punishment is unlikely to promote cooperation from targets [18,60]: an extension of this prediction might be that morally illegitimate punishment makes players more likely to defect and to retaliate. Supporting this idea, a recent study that incorporated power asymmetries in a two-player game where senior workers could exploit junior partners (by suggesting that they contribute larger investments and punishing them if they failed to obey) showed that junior workers did not obey strong partners when they knew that they were being exploited [46]. Conversely, when junior workers only had incomplete information about how much the other player earned, they were more likely to comply with the senior worker's suggestion [46]. Theoretical work has shown that in multi-player games, punishment may be cost-effective if multiple individuals within the group collectively punish defectors [59]. Such collective punishment may also be perceived to be more legitimate than punishment from a single group member because it is more likely to be in the collective interest, rather than in the interest of a selfish individual. Although, punishment in this study was decentralized, the legitimacy of punishment is also likely to be important under centralized punishment regimes. For example, punishment is likely to be more effective at promoting cooperation when centralized authorities have been legitimately elected; rather than chosen at random [61]. In light of these results and the results of our study, we suggest that further work to explore the moral assessment of punishment in different circumstances would be very helpful to understand why punishment sometimes promotes and sometimes undermines cooperation. It is important that this future work should collect data on both players’ behavioural responses as well as their subjective evaluations of punishment.

In general, weak players opted out more than strong players and both player types opted out more when faced with a strong partner. Although, theoretical studies have suggested that cooperation and punishment is more likely to occur and persist if players have the option to opt out [62,63], these models assumed that all players were equal in strength, used multi-player rather than two-player games and permitted only a small number of behavioural strategies. In our study, players were more likely to opt out of rounds if they were previously punished than when they were not punished. This suggests that both player types occasionally chose to avoid further punishment by withdrawing from the game rather than by switching to cooperation. Punishment may be more effective at promoting cooperation when players are unable to opt out of rounds and can therefore only avoid further punishment by switching to cooperation.This hypothesis could be tested by repeating this experiment but excluding the option for players to opt out of rounds.

Weak and strong players were also more likely to opt out of rounds if their partner defected than if their partner cooperated in the previous round. This suggests that players used opting out either to avoid defectors or to signal their disapproval with the defector's action. However, opting out appeared to decrease the probability that a defecting partner would switch to cooperation (see ESM for analysis).

To summarise, we found no evidence that punishment promoted cooperation in this two-player game and in fact punishment use sometimes had a detrimental effect on cooperation. Future work could explore whether these results stem from using a binary response term for cooperation and whether punishment might be more effective where investments are continuous. We also propose that understanding the moral assessment of punishment will be crucial for predicting when it will promote and when it will undermine cooperation.

## Supporting Information

S1 Supporting Information Experimental data. Columns are as follows: Partner coop prev = whether the players partner cooperated (yes) or defected (no) in the previous round; Player C after D = 1 if player cooperated and 0 if player defected in round n + 1 after defecting in round n; Player P in response to C = 1 if the player punished and 0 if the player didn't punish in response to a cooperative partner; Partner P after D prev = 1 if the partner punished and 0 if the player didn't punish in response to a defecting player in the previous round; Player opt out after D = 1 if player opted out and 0 if player didn't opt out of round n + 1 after defecting in round n; Player P in response to D = 1 if the player punished and 0 if the player didn't punish in response to a defecting partner; Partner opt out prev = 1 if players partner opted out in the previous round and 0 if player did not opt out in the previous round; Player C after D 2 = If partner did not opt out of round n + 1; 1 if player cooperated and 0 if player defected in round n + 1 after defecting round n. If partner opted out of round n + 1: 1 if player cooperated and 0 if player defected in round n + 2 after defecting round n.
(CSV)

S2 Supporting Information Supporting Text, Tables & Figures. Experimental instructions, comprehension questions, demographic questions, analysis of players’ response to partner opting out, supplementary tables, supplementary figures & R code used to fit generalised linear mixed models.
(DOC)
