# Supplementary material for: The Effect of Power Asymmetries on Cooperation and Punishment in a Prisoner’s Dilemma Game
Source: PLoS One. 2015 Jan 28;10(1):e0117183. doi: 10.1371/journal.pone.0117183 (PMC4309618; doi:10.1371/journal.pone.0117183)
Supplement: S2 Supporting Information — Experimental instructions, comprehension questions, demographic questions, analysis of players’ response to partner opting out, supplementary tables, supplementary figures & R code used to fit generalised linear mixed models. (DOC) [file pone.0117183.s002.doc]

**Electronic supplementary materials**

**Table of contents**

|  |  |  |
| --- | --- | --- |
| 1 | Experimental instructions | p. 2 - 4 |
| 2 | Comprehension questions | p. 5 |
| 4 | Demographic questions | p. 5 |
| 5 | Analysis of players’ response to partner opting out | p. 6 |
| 6 | Supplementary tables | p.7 - 11 |
| 7 | Supplementary figures | p. 11 - 12 |
| 8 | R code used to fit generalised linear mixed models | p. 12 - 16 |

**1. Experimental instructions**

Participants were given a printed copy of the instructions below. They were given 15 minutes to read through the instructions before attempting the comprehension questions. Participants were free to refer back to the instructions whilst completing the comprehension questions and throughout the experiment.

**Welcome and thank you for volunteering for this experiment.**

Please be quiet during the entire experiment. **Do** **not talk to your neighbours** and do not try to look at their screens. If you have any questions, please raise your hand. We will come to you and answer it privately.

This experiment is about decision-making. **You will be randomly assigned the role as either a “type 1” or a “type 2” player.** You will keep this role during the entire experiment. You will play a two-player game **twice.** Each game will last for a predetermined number of rounds **(between 20 and 100).**

In each game you **will be randomly matched with a different person in the room.** You will play with the same person for the duration of each game. Therefore, you will play with two people in total. In one game you will play with someone of the same type as yourself and in one game you will play someone of a different type to yourself. **You will remain anonymous throughout the experiment** and will be identified only by the name in the top left corner of your computer screen.

**Before playing the games you will answer some questions about the experiment.** The purpose of this is to make sure that everybody fully understands the rules of the experiment before we start. So please make sure you read these instructions carefully.

Depending on your decisions and the decisions made by the other player in each of the two games you play, you will be able to earn a considerable amount of money. The scores you receive in the game will be given in units **(1 unit= 6p).**

Everyone **will receive a show-up fee of £5.** In addition, you will be given **an additional 75 units (£4.50) to play with at the start of each game**. Depending on the decisions made by you and the other player during each game you will gain or lose units from this initial amount. After the experiment has finished, your units will be converted to real money. You will be paid the show-up fee and the money that you earn. In total this could be as much as £32.

**Payment:** Payment will happen after both games have been played.

**The Game:**

**Each round will be** **split into two steps. In each step you will be asked to make a decision. The decisions you make will affect the score you and the other player receives for that round.** There is a time limit of **15 seconds** on each step (shown in the top right corner of the screen). It is important you make a choice within this time, if not **a default choice will be picked for you** and you will move on to the next step.

**Each of these steps will now be explained to you in detail:**

***Step 1:***

Both you and the other player **simultaneously** choose between the options, **“A”, “B” or “Do not participate in this round”.**

If **you choose “A”** then **you** will get **−1 unit**, whereas the **other player** will get +**2 units**.

**If you choose “B” then you will get +1 unit, whereas the other player will get −1 unit.**

If either player chooses “Do not participate in this round” **both you and the other player will skip the second step and move to step 1 of the next round. Neither player will gain or lose any units in this entire round. The next round will begin as normal.**

***Step 2: If either player chose “Do not participate in this round” in step 1 then this round is skipped. Otherwise,*** you and the other player are **presented with each other’s choices and scores from step 1.** You must both then **decide** whether you would or would not like to reduce the other player's income at a cost to yourself by choosing between **options “C”** or **“D”, respectively.**

Option “C” = reduce the other player's income at a cost of 1 unit to yourself.

Option “D” = do nothing (neither you nor the other player will gain or lose any points).

**The number of units that the other player’s income is reduced by when you choose option “C” varies depending upon what “type” of player you were assigned as at the start of the experiment.**

**Type 1 player:** **If you choose “C” then you will lose 1 unit, and the other player will lose 1 unit.**

**Type 2 player: if you choose “C” you will lose 1 unit, and the other player will lose 4 units.**

After you have made a decision, you and the other player are **presented with each other’s choices and incomes from step 2** as well as your **overall incomes** for the round. You will also be told your **total score** for the current game.

Your overall income in each step **is determined by the addition of the income from both your decision and the other player's decision***.*

Some examples are given below;

***Step1*:**

If **both you and the other player choose “A”** then you will get **+1** (-1 from yourself, +2 from the other player = +1 total).

If **both you and the other player choose “B”** then you will get **0** (+1 from yourself, -1 from the other player= 0 total).

If **you choose “A”**, and the other player **chooses “B”** then **you will** get **–2** (-1 from yourself, -1 from the other player = -2 total).

If **you choose “B” and the other player chooses “A” then you** get **+3** (+1 from yourself, +2 from the other player = +3 total).

***Step 2:*** *If* ***either player chose “Do not participate in this round” in step 1 then this step is skipped.***

If you **both you and the other player choose “C”** then you will get **-2 if the other player is “type 1” player** (-1 from yourself, -1 from the other player = -2 total) **and -5 if the other player is “type 2” player** (-1 from yourself, -4 from the other player= -5 total).

If you **both you and the other player choose “D”** then you will **both get 0** (0 from yourself, 0 from the other player =0 total).

If **you choose “C”**, and the other **player chooses “D”** then **you** get -1 (-1 from yourself, 0 from the other player = -1 total).

If you **choose “D”**, and **the other player chooses “C”** then **you** get **-1 if the other player is a “type 1” player** (0 from yourself, -1 from the other player = -1 total) **or -4 if the other player is a “type 2” player** (0 from yourself -4 from the other player = -4 total).

**Your income for the round will be determined by the addition of your income from step 1 and step 2.**

**The total number of units that you have at the end of these games will determine how much money you have earned.** Therefore, the additional money you and the other player each earn depends on which options you both choose. However, **the final scores of the other players do not matter for your earnings.**

If your **total score** drops **to 0 units or below**, you **will not be able to play** for the remaining rounds of the game of the **current game**.

At the end of both games, your total earnings will be computed. If you finish with a total score of **0 over the two games**, you will walk away with just the £5 show up fee. If you have a total score **above 0**, you will earn extra money at the exchange rate of 1 unit= 6p. The maximum extra amount that you can earn will be **£27**.

We will distribute a questionnaire at the end of the experiment that will ask some basic information about you.

Take your time to read through the instructions again. If you have any questions, please raise your hand. In a few minutes we will begin the questionnaire followed by the games.

**2. Comprehension questions**

All participants were required to answer the following comprehension questions before playing the game. If they answered a question incorrectly they were shown the correct answer. The possible answers are shown in parentheses.

1. Each game will last a predetermined number of rounds, between what? (10-200 / 20-100 / 0-50)

2. How many games will you play in this experiment? (1 / 5 / 2)

3. If you finish the experiment with a total score of 100 units, how much money will you earn on top of the £5 show up fee (1 unit = 6p)? (£6 / £10 / £80)

4. If your total score drops to 0 or below in a game what will happen? (You will lose your show up fee / You will have to leave the room / You will not be able to play the remaining rounds of the current game)

5. If you chose option "B" and the other player chooses option "A", how many units will you get in step 1? (3 / 1 / 10)

6. If both you and the other player choose option "A", how many units will you get in step 1? (1 / 15 / 1)

7. If you are a "type 1" player and choose option "C" in step 2, how many units will be deducted from the other players’ income? (1 / 3 / 5)

8. If both you and the other player are "type 2" players and you both choose option "C", how many units will you get in step 2? (-1 / -5 / 2)

9. If both you and the other player are "type 1" players, you both choose option "B" in step 1 and both choose option "D" in step 2, how many units will you get in that round? (0 / 2 / -5)

**3. Demographic questions**

After the game had finished, all participants were required to answer the following demographic questions. Responses to these demographic questions can be found in the supplementary data.

1. What is your gender?

2. How old are you?

3. What is your country of origin?

4. What is your subject of study?

**5. Analysis of players’ response to partner opting out**

We asked how defecting players responded to their partner opting out (rather than punishing) in the previous round. Data were restricted to instances where players had defected in round *n* (i.e. the effect of antisocial punishment on target behaviour was not measured; n = 1394 rounds). Generalised linear mixed models (GLMMs) with model averaging were used for this analysis (as described in the main text). The response term used encoded players responses to their partner opting out of the previous round (If partner did not opt out of round n + 1: set as 1 if player cooperated and 0 if player defected in round n + 1 after defecting round n. If partner opted out of round n + 1: set as 1 if player cooperated and 0 if player defected in round n + 2 after defecting round n). The explanatory variables included for this model were: whether the partner opted out in the previous round (Partner opted out; yes), the player’s type (strong), the game type (asymmetric) and all 2-way and 3-way interactions

Regardless of player type or game type, players were slightly less likely to cooperate if their partner opted out in the previous round. However, the confidence intervals for this term include zero, meaning that this effect is weak (Table S5).

**6. Supplementary tables**

| **Parameter** | **Weak** | **Strong** |
| --- | --- | --- |
| Age | Mean = 22.2 ± 0.65 | Mean = 21.8 ± 0.44 |
|  | Median = 21 | Median = 21 |
|  | IQR = 24 – 33 | IQR = 23 – 33.25 |
|  | Range = 20 - 23 | Range = 20 - 23 |
|  |  |  |
|  |  |  |
| Gender (n) | Females = 36 | Females = 27 |
|  | Males = 24 | Males = 33 |
|  |  |  |
| Country of Origin (n) | Canada = 1 | China = 10 |
|  | China = 10 | Czech Republic = 1 |
|  | Cyprus = 1 | Greece = 1 |
|  | France = 1 | Hong Kong = 5 |
|  | Germany = 1 | Hungary = 1 |
|  | Greece = 2 | Malaysia = 5 |
|  | Hong Kong = 6 | Mauritius = 1 |
|  | India = 4 | Nigeria = 1 |
|  | Iraq = 2 | Pakistan = 1 |
|  | Italy = 2 | Romania = 2 |
|  | Malawi = 1 | Singapore = 8 |
|  | Malaysia = 3 | Sri Lanka = 1 |
|  | Pakistan = 1 | Sudan = 1 |
|  | Poland = 3 | Taiwan = 1 |
|  | Romania = 1 | United Kingdom = 17 |
|  | Singapore = 4 | United States = 1 |
|  | United Kingdom = 11 | Vietnam = 2 |
|  | United States = 2 |  |
|  | Undisclosed = 1 |  |
|  |  |  |
| Subject Studied (n) | Anthropology = 1 | Archaeology = 1 |
|  | Architecture = 2 | Biochemical Engineering = 1 |
|  | Biochemistry = 1 | Biochemistry = 2 |
|  | Biology = 1 | Biotechnology = 2 |
|  | Biomedical Sciences = 5 | Civil Engineering = 1 |
|  | Biotechnology = 1 | Classics = 1 |
|  | Chemical Engineering = 1 | Economics = 9 |
|  | Chemistry = 1 | Engineering = 1 |
|  | Cognitive Neuroscience = 1 | English Linguistics = 1 |
|  | Computer Science = 1 | Financtial Risk Management = 1 |
|  | Economics = 10 | Fine Art = 1 |
|  | Economics & Business = 1 | History = 1 |
|  | Egyptian Archaeology = 1 | Human Science = 1 |
|  | Engineering = 3 | Languages = 1 |
|  | English = 1 | Law = 5 |
|  | Genetics = 1 | Mathematics = 5 |
|  | History of Art = 2 | Medicene = 4 |
|  | Hunanities = 1 | Management = 1 |
|  | International Planning = 1 | Natural Science = 1 |
|  | International Public Policy = 1 | Neuroscience = 2 |
|  | Italian & Art History = 1 | Nutrition = 1 |
|  | Law = 1 | Pharmacy = 3 |
|  | Hispanic Culture = 1 | Physics and Biology = 1 |
|  | Librarianship = 1 | Politics = 1 |
|  | Medicine = 1 | Psychology = 3 |
|  | Global Health = 1 | Security Services = 1 |
|  | Managment = 1 | Speech Science = 2 |
|  | Natural Sciences = 2 | Statistics = 1 |
|  | Neuroscience = 2 | Physics = 1 |
|  | Pharmacy = 3 | Urban Design = 1 |
|  | Philosophy = 1 | Urban Planning = 2 |
|  | Psychology = 2 | Viking Studies = 1 |
|  | Russian and Italian = 1 |  |
|  | Statistics = 1 |  |
|  | Physics = 1 |  |
|  | Urban Design = 1 |  |
|  | Urban Planning = 2 |  |

**Table A.** Information on mean, median values and sample sizes for demographic data.

| Parameter | Effect size | SE | Confidence Interval | Importance |
| --- | --- | --- | --- | --- |
| Intercept | -1.51 | 0.14 | (-1.78, -1.24) |  |
| Player type (strong) | 0.62 | 0.28 | (0.09, 1.17) | 1.00 |
| Game type (asymmetric) | 0.17 | 0.21 | (-0.23, 0.57) | 1.00 |
| Partner cooperated (yes) | 1.94 | 0.16 | (1.62, 2.27) | 1.00 |
| Partner punished (yes) | 0.10 | 0.22 | (-0.34, 0.54) | 1.00 |
| Player type x Game type | -0.96 | 0.42 | (-1.78, -1.13) | 1.00 |
| Player type x Partner cooperated | 0.31 | 0.33 | (-0.36, 0.94) | 1.00 |
| Player type x Partner punished | -0.67 | 0.39 | (-1.43, 0.09) | 1.00 |
| Game type x Partner cooperated | -0.62 | 0.34 | (-1.28, 0.04) | 1.00 |
| Game type x Partner punished | 0.80 | 0.46 | (-0.09, 1.69) | 0.70 |
| Partner cooperated x Partner punished | -1.02 | 0.38 | (-1.76, -0.28) | 1.00 |
| Player type x Game type x Partner cooperated | 1.58 | 0.67 | (0.25, 2.90) | 1.00 |
| Player type x Game type x Partner punished | 0.79 | 0.77 | (-0.72, 2.30) | 0.27 |
| Game type x Partner cooperated x Partner punished | 1.10 | 0.74 | (-0.35, 2.56) | 0.52 |

**Table B.** Effect sizes, unconditional standard errors, confidence intervals and relative importance for parameters included in the top models investigating players' responses to being punished for defecting in the previous round (player continued to defect = 0, player switched to cooperate = 1).

| Partner type | Parameter | Effect size | SE | Confidence Interval | Importance |
| --- | --- | --- | --- | --- | --- |
| Strong | Intercept | -3.34 | 0.84 | (-7.45, -2.18) |  |
|  | Punished (yes) | 1.96 | 0.73 | (0.58, 3.57) | 1.00 |

**Table C.** Effect sizes, unconditional standard errors, confidence intervals and relative importance for parameters included in the top models investigating whether a player retaliated against a punitive partner (player did not punish cooperative partner = 0, player did punish cooperative partner = 1).

| Parameter | Effect size | SE | Confidence Interval | Importance |
| --- | --- | --- | --- | --- |
| Intercept | -2.60 | 0.18 | (-2.95, -2.25) |  |
| Player type (strong) | -1.10 | 0.33 | (-1.74, -0.46) | 1.00 |
| Game type (asymmetric) | -0.14 | 0.20 | (-0.54, 0.26) | 1.00 |
| Partner punished | 0.94 | 0.20 | (0.55, 1.32) | 1.00 |
| Partner cooperated | -1.04 | 0.24 | (-1.51, -0.57) | 1.00 |
| Player type x Game type | -1.36 | 0.45 | (-2.24, -0.47) | 1.00 |
| Player type x Partner cooperated | 0.56 | 0.45 | (-0.33, 1.44) | 0.39 |
| Game type x Partner cooperated | -0.44 | 0.43 | (-1.28, 0.39) | 0.35 |
| Partner cooperated x Partner punished | 0.38 | 0.44 | (-0.49, 1.24) | 0.31 |

**Table D.** Effect sizes, unconditional standard errors, confidence intervals and relative importance for parameters included in the top models investigating whether players opted out (player did not opt out = 0, player opted out = 1).

| Parameter | Effect size | SE | Confidence Interval | Importance |
| --- | --- | --- | --- | --- |
| Intercept | 1.35 | 0.00 | (-1.35, -1.35) |  |
| Game type (asymmetric) | -0.17 | 0.00 | (-0.17, -0.16) | 1.00 |
| Player type (strong) | 1.12 | 0.00 | (1.12, 1.13) | 1.00 |
| Game type x Player type | -1.44 | 0.00 | (-1.45, -1.44) | 1.00 |
| Partner opted out (yes) | -0.06 | 0.03 | (-0.06, -0.06) | 0.27 |

**Table E.** Effect sizes, unconditional standard errors, confidence intervals and relative importance for parameters included in the top models investigating how players that defected in round n responded to their partner opting out of round n + 1 (player continued to defect = 0, player switched to cooperate = 1).

**7. Supplementary figures**

**
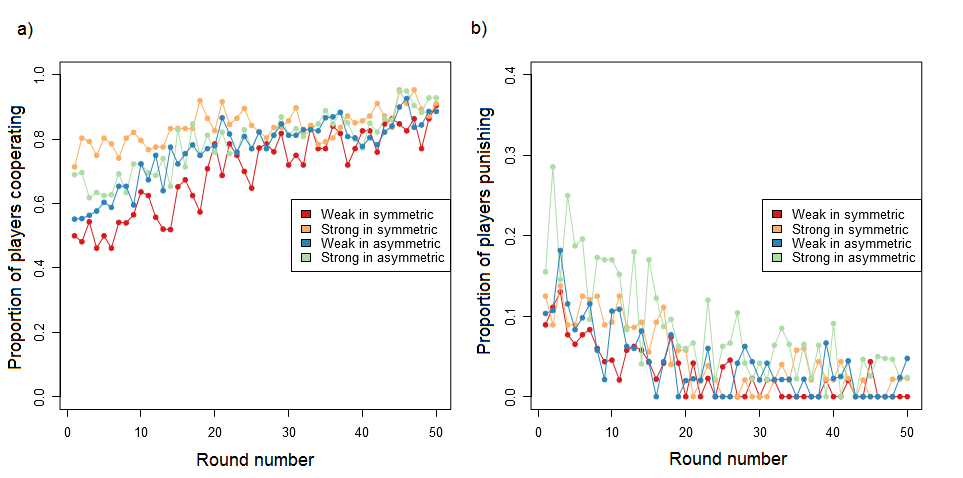
**

**Figure A.** Scatter plot showing the mean proportion of players that **a)** cooperated and **b)** punished their partner, according to whether they were weak or strong and whether they were in a symmetric or asymmetric game. Rounds where either player opted out or was bankrupt were excluded.


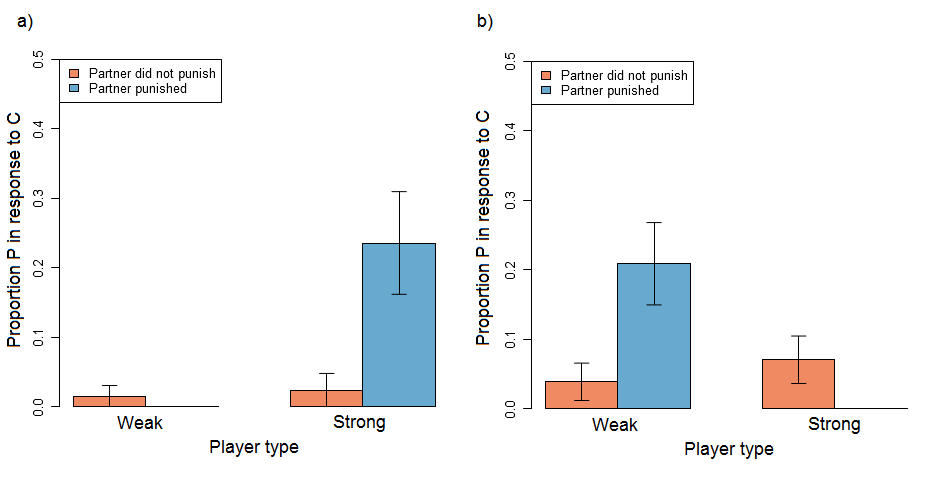


**Figure B.** Barplot showing the mean proportion of players in **a)** symmetric and **b)** asymmetric games that punished their partner for cooperating, according to whether they were weak or strong and whether they were punished by their partner in the previous round. Data were restricted to instances in which the player defected in the previous round and their partner cooperated in the current round. Rounds where either player opted out or was bankrupt were also excluded. Error bars represent standard errors. Thus, red bars represent antisocial punishment whereas blue bars can be interpreted as retaliation for punishment previously received. Plots are generated from raw data.

**8. R code used to fit generalised linear mixed models**

#Analysis (i)

data<-read.csv(file="Data.csv",head=TRUE,sep=",")

data$Player.type<-relevel(data$Player.type, "Weak")

data$Game.type <- relevel(data$Game.type, "Symmetric")

data<-data[complete.cases(data$Player.cooperated),]

global.model<-glmer(Player.cooperated~Game.type*Player.type+(1|Subject.ID),data=data,family="binomial",na.action = "na.fail")

global.model

stdz.model<-standardize(global.model,standardize.y = FALSE)

model.set<-dredge(stdz.model)

top.models<-get.models(model.set, subset = delta<2)

mi<-stdz.model

summary(mi)

confint(mi)

#Analysis (ii)

data<-read.csv(file="Data.csv",head=TRUE,sep=",")

data$Player.type <- relevel(data$Player.type, "Weak")

data$Game.type<-relevel(data$Game.type, "Symmetric")

data<-data[complete.cases(data$Player.cooperated),]

data<-data[complete.cases(data$Player.P.in.response.to.D),]

global.model<-glmer(Player.P.in.response.to.D~Player.cooperated*Player.type*Game.type+(1|Subject.ID),data=data,family="binomial",na.action = "na.fail")

stdz.model<-standardize(global.model,standardize.y = FALSE)

model.set<-dredge(stdz.model)

top.models<-get.models(model.set, subset = delta<2)

mii<-model.avg(top.models)

summary(mii)

confint(mii)

#Analysis (iii)

data<-read.csv(file="Data.csv",head=TRUE,sep=",")

data$Player.type<-relevel(data$Player.type, "Weak")

data$Game.type<-relevel(data$Game.type, "Symmetric")

data<-data[complete.cases(data$Player.C.after.D),]

data.player.weak<-data[data$Player.type=="Weak",]

data.player.strong.partner.coop<-data[data$Player.type=="Strong"&data$Partner.coop.prev=="Yes",]

data.player.strong.partner.def<-data[data$Player.type=="Strong"&data$Partner.coop.prev=="No",]

#a.1

global.model<-glmer(Player.C.after.D~ Partner.P.after.D.prev*Game.type*Partner.coop.prev

+(1|Subject.ID),data=data.player.weak,family="binomial",na.action = "na.fail")

stdz.model<-standardize(global.model,standardize.y = FALSE)

model.set<-dredge(stdz.model)

top.models<-get.models(model.set, subset = delta<2)

model.avg(top.models)->miiia1

summary(miiia1)

confint(miiia1)

#b.1

global.model<-glmer(Player.C.after.D~ Partner.P.after.D.prev*Game.type

+(1|Subject.ID),data=data.player.strong.partner.def,family="binomial",na.action = "na.fail")

stdz.model<-standardize(global.model,standardize.y = FALSE)

model.set<-dredge(stdz.model)

top.models<-get.models(model.set, subset = delta<2)

model.avg(top.models)->miiib1

summary(miiib1)

confint(miiib1)

#b.2

global.model<-glmer(Player.C.after.D~Partner.P.after.D.prev*Game.type

+(1|Subject.ID),data=data.player.strong.partner.coop,family="binomial",na.action = "na.fail")

stdz.model<-standardize(global.model,standardize.y = FALSE)

model.set<-dredge(stdz.model)

top.models<-get.models(model.set, subset = delta<2)

top.models

miiib2<-stdz.model

summary(miiib2)

confint(miiib2)

#Analyis (iv)

data<-read.csv(file="Data.csv",head=TRUE,sep=",")

data$Game.type <- relevel(data$Game.type, "Symmetric")

data<-data[complete.cases(data$Player.P.in.response.to.C),]

data.partner.strong<-data[data$Partner.type=="Strong",]

#b

global.model<-glmer(Player.P.in.response.to.C~ Partner.coop.prev*Partner.P.after.D.prev*Player.type+(1|Subject.ID),data=data.partner.strong,family="binomial",na.action = "na.fail")

stdz.model<-standardize(global.model,standardize.y = FALSE)

model.set<-dredge(stdz.model)

top.models<-get.models(model.set, subset = delta<2)

top.models

global.model<-glmer(Player.P.in.response.to.C~ Partner.P.after.D.prev+(1|Subject.ID),data=data.partner.strong,family="binomial",na.action = "na.fail")

mivb<-standardize(global.model,standardize.y = FALSE)

summary(mivb)

#Analysis (v)

data<-read.csv(file="Data.csv",head=TRUE,sep=",")

data$Player.type <- relevel(data$Player.type, "Weak")

data$Game.type <- relevel(data$Game.type, "Symmetric")

data<-data[complete.cases(data$Player.opt.out.after.D),]

data<-data[complete.cases(data$Partner.P.after.D.prev),]

global.model<-glmer(Player.opt.out.after.D~Game.type*Player.type*Partner.coop.prev+Game.type*Player.type*Partner.P.after.D.prev+Game.type*Partner.coop.prev*Partner.P.after.D.prev+Player.type*Partner.coop.prev*Partner.P.after.D.prev

+(1|Subject.ID),data=data,family="binomial",na.action = "na.fail")

stdz.model <- standardize(global.model,standardize.y = FALSE)

model.set <- dredge(stdz.model)

top.models <- get.models(model.set, subset = delta<2)

mv<-model.avg(top.models)

confint(mv)

#Supplementary analysis

data<-read.csv(file="Data.csv",head=TRUE,sep=",")

data$Player.type<-relevel(data$Player.type, "Weak")

data$Game.type<-relevel(data$Game.type, "Symmetric")

data<-data[complete.cases(data$Player.C.after.D.2),]

global.model<-glmer(Player.C.after.D.2~Partner.opt.out.prev+Player.type*Game.type+(1|Subject.ID),data=data,family="binomial",na.action = "na.fail")

stdz.model<-standardize(global.model,standardize.y = FALSE)

global.model

model.set<-dredge(stdz.model)

top.models<-get.models(model.set, subset = delta<2)

mSi<-model.avg(top.models)

confint(mSi)

summary(mSi)
